# Unraveling the mystery of auxin-promoting femaleness in cucurbits

**DOI:** 10.1093/hr/uhaf354

**Published:** 2025-12-24

**Authors:** Liu Xiaofeng, Zhang Zhonghua, Sun Jinjing

**Affiliations:** College of Horticulture, Qingdao Agricultural University, Qingdao 266109, China; College of Horticulture, Qingdao Agricultural University, Qingdao 266109, China; State Key Laboratory of Vegetable Biobreeding, Institute of Vegetables and Flowers, Chinese Academy of Agricultural Sciences, Beijing 100081, China

## Abstract

The female flower gives rise to the fruit/seed and thus directly affects crop yield in unisexual plants. Both ethylene and auxin promote femaleness in cucurbits. However, how auxin regulates sex determination has been an open question over half a century. The recent publication identified auxin response factor *CsARF3* as a crucial player in auxin-promoting femaleness, and revealed a reciprocal relationship between auxin and ethylene during female flower determination.

Unisexual flower development is one of the most significant evolutionary landmarks in angiosperms [1]. Cucurbits have been served as ideal models for sex determination studies, in which unisexual flowers are produced by selective arrest of pistil or stamen development at specific stages of floral buds [2]. Over the past two decades, impressive progress has been made about how ethylene regulates sex determination in cucurbits. Both *ACS11* and *ACS2/ACS7* encode two aminocyclopropane-1-carboxylic acid (ACC) synthases that function as rate-limiting steps in ethylene biosynthesis [3, 4]. *ACS11* exhibits carpel-specific expression pattern in female flowers. WIP domain protein 1 (WIP1), a C2H2 zinc-finger transcription factor, suppresses carpel development by directly repressing the expression of the YABBY family gene *CRC* [5, 6]. Ethylene biosynthesis initiated by ACS11 suppresses *WIP1* via the canonical ETHYLENE INSENSITIVE2/3/ETHYLENE-INSENSITIVE3-LIKE 1 (EIN2/EIN3/EIL1) ethylene signaling cascade, thereby establishing female developmental fate [5, 7, 8]. Concurrently, ACS2 activates the ETR1-EIN3/EIL1 ethylene signaling module in stamen primordia, triggering a stamen-suppressing gene *Homeobox protein 40* to govern androecium programming [7]. Moreover, the *Female* (*F*) locus conferring gynoecy, which encodes the ACC synthase ACS1G, was identified in cucumber by Zhang *et al.* [6]. The ethylene burst produced by F locus and ACO2 bypasses ACS11 to establish the dominant gynoecious phenotype.

As early as 1950s, auxin was proposed to promote femaleness in cucumber, but the molecular mechanism by which auxin regulates sex determination has remained elusive [9]. Zhang and her team first found that the auxin response factor *CsARF3* plays a key role in female flower determination (Fig. 1A). *CsARF3* mutation results in producing only male flowers, whereas its overexpression increases the proportion of female flowers. *CsARF3* promotes femaleness by directly stimulating *Cucumis sativus Shoot Meristemless* expression and repressing *CsWIP1* expression in cucumber. Auxin treatment failed to rescue female flower production in the *Csarf3* mutant, suggesting that CsARF3-mediated signaling is essential for femaleness in cucumber. These results greatly advance our understanding of how auxin regulates sex determination in plants.

**Figure 1:**
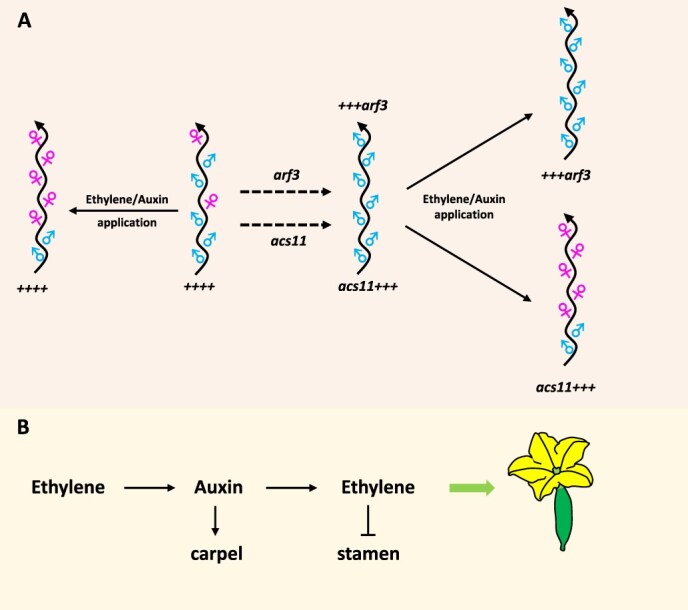
Ethylene-promoting femaleness depends on ARF3-mediated auxin signaling pathway in cucumber; A. ARF3 is essential for ACS11-mediated ethylene promotion of female flower development; the symbols ‘++++’ represent the dominant alleles of the *ACS11*, *WIP1*, *ACS2*, and *ARF3* genes, respectively; and B. auxin and ethylene reciprocally promote femaleness in cucumber.

The dominate *ACS1G* is expressed at very early stages of floral development to generate an ethylene burst and the gynoecy phenotype in cucumber. Zhang and her team also showed that *CsARF3* mutation could arrest ACS1G-inducing female flower development, indicating that CsARF3 acts genetically downstream of CsACS1G to control carpel development in cucumber. Moreover, ethephon application failed to induce female flowers in *Csarf3* mutants, while auxin treatment restored female flower development in the ethylene biosynthesis mutant *Csacs11* bearing only male flowers. Thus, ethylene-mediated female induction is dependent on auxin at the early stage. *ACS2/ACS7* is highly expressed in the carpel primordia of female flowers rather than male flowers to inhibit stamen development. The expression of *CsACS2* was markedly reduced in *Csarf3* and elevated in *CsARF3*-OE lines, indicating that CsARF3-induced signaling or carpel formation may be a prerequisite for *CsACS2*-mediated ethylene biosynthesis and stamen inhibition. These data demonstrate that ethylene and auxin exhibit a reciprocal relationship to orchestrate sex determination [10].

Collectively, these findings uncover a novel mechanism of auxin regulating sex determination in flowering plants and elucidate a long-sought link between auxin and ethylene in this process, in which ethylene promotes carpel formation through auxin at the early stage, and then auxin boosts ethylene biosynthesis in carpel to inhibit stamen development (Fig. 1B). This work significantly advances our understanding of the fundamental mechanisms of hormone-mediated sex determination in plants. Meanwhile, given the close association between sex types and crop yield, this report represents significant importance in plant breeding.

## References

[1] Karron JD, Ivey CT, Mitchell RJ. et al. New perspectives on the evolution of plant mating systems. Ann Bot. 2012;109:493–503 22210849 10.1093/aob/mcr319 PMC3278297

[2] Bai SL, Peng YB, Cui JX. et al. Developmental analyses reveal early arrests of the spore-bearing parts of reproductive organs in unisexual flowers of cucumber (*Cucumis sativus* L.). Planta. 2004;220:230–40 15290297 10.1007/s00425-004-1342-2

[3] Boualem A, Fergany M, Fernandez R. et al. A conserved mutation in an ethylene biosynthesis enzyme leads to Andromonoecy in melons. Science. 2008;321:836–8 18687965 10.1126/science.1159023

[4] Boualem A, Troadec C, Camps C. et al. A cucurbit androecy gene reveals how unisexual flowers develop and dioecy emerges. Science. 2015;350:688–91 26542573 10.1126/science.aac8370

[5] Martin A, Troadec C, Boualem A. et al. A transposon-induced epigenetic change leads to sex determination in melon. Nature. 2009;461:1135–8 19847267 10.1038/nature08498

[6] Zhang S, Tan FQ, Chung CH. et al. The control of carpel determinacy pathway leads to sex determination in cucurbits. Science. 2022;378:543–9 36378960 10.1126/science.add4250

[7] Rashid D, Devani RS, Rodriguez-Granados NY. et al. Ethylene produced in carpel primordia controls *CmHB40* expression to inhibit stamen development. Nat Plants. 2023;9:1675–87 37653338 10.1038/s41477-023-01511-z

[8] Huang H, Zhang S, Choucha FA. et al. Harbinger transposon insertion in ethylenesignaling gene leads to emergence of newsexual forms in cucurbits. Nat Commun. 2024;15:4877 10.1038/s41467-024-49250-9 PMC11161486 38849342

[9] Galun E . The role of auxins in the sex expression of the cucumber. Physio Plantarum. 1959;12:48–61

[10] Han L, Li M, Li C. et al. ARF3-mediated auxin signaling is essential for sex determination in cucumber. Science. 2025;eadv2006 10.1126/science.adv2006 41379936

